# Comparative Analysis of the Progesterone Receptor Interactome in the Human Ovarian Granulosa Cell Line KGN and Other Female Reproductive Cells

**DOI:** 10.1002/pmic.202400374

**Published:** 2025-05-13

**Authors:** Natalie J. Foot, Doan T. Dinh, Samantha J. Emery‐Corbin, Jumana M. Yousef, Laura F. Dagley, Darryl L. Russell

**Affiliations:** ^1^ Robinson Research Institute, School of Biomedicine, Faculty of Health & Medical Sciences University of Adelaide Adelaide Australia; ^2^ Advanced Technology and Biology Division Walter and Eliza Hall Institute Parkville Victoria Australia; ^3^ Department of Medical Biology University of Melbourne Melbourne Victoria Australia

## Abstract

The nuclear steroid hormone receptor progesterone receptor (PGR) is expressed in granulosa cells in the ovarian follicle in a tightly regulated pattern in response to the surge of luteinizing hormone (LH) that stimulates ovulation. PGR plays a critical role in mediating ovulation in response to LH, however, the mechanism for this is still unknown. We performed immunoprecipitation‐mass spectrometry using the KGN human granulosa cell line expressing the primary PGR isoforms PGR‐A or PGR‐B, to identify novel interacting proteins that regulate PGR function in these ovary‐specific target cells. Proteomic analysis revealed protein interactions with both PGR isoforms that were gained (e.g., transcriptional coactivators) or lost (e.g., chaperone proteins) in response to the PGR agonist R5020. Additionally, isoform‐specific interactions, including different families of transcriptional regulators, were identified. Comparison with published datasets of PGR‐interacting proteins in human breast cancer cell lines and decidualized endometrial stromal cells demonstrated a remarkable number of tissue‐specific interactions, shedding light on how PGR can maintain diverse functions in different tissues. In conclusion, we provide a comprehensive novel dataset of the PGR interactome in previously unstudied ovarian cells and offer new insights into ovary‐specific PGR transcriptional mechanisms.

**Abbreviations**
KGN: ovarian granulosa cell tumor cell line
LH: luteinizing hormone
PGR: progesterone receptor

## 1 Introduction

Progesterone receptors (PGRs) are pivotal modulators of a broad range of physiological processes, primarily within the reproductive system, mammary gland and central nervous system. These nuclear receptors operate as ligand‐dependent transcription factors that modulate gene expression in response to the steroid hormone progesterone. The activity of PGR is intricately regulated not only by its ligands but also by a myriad of interacting proteins that modulate its stability, localization and transcriptional activity. Understanding these interactions is critical for elucidating the complex mechanisms of PGR action in both normal physiology and disease.

PGR exists in two main isoforms, PGR‐A and PGR‐B, which are transcribed from a single gene through alternative promoter usage [1, 2, 3, 4]. These isoforms are identical except for an extended N‐terminal region containing an AF3 protein interaction domain in PGR‐B, and exhibit distinct functional roles, with PGR‐B typically acting as a more potent transcriptional activator than PGR‐A [5]. The functional diversity of PGR is further expanded through its interaction with various co‐regulators and signaling molecules, which fine‐tune its transcriptional activity and cellular responses to Progesterone. These interactions can enhance or inhibit PGR‐mediated transcription, influence PGR stability, and affect receptor localization. Coactivators, such as the steroid receptor coactivator (SRC or NCOA) family, enhance PGR activity by facilitating the recruitment of transcriptional machinery to target gene promoters [6]. Chaperone proteins such as HSP90 and FKBP5 are essential for the proper folding, stability, and function of PGRs [7]. Additionally, PGR interacts with various signaling proteins, which modulate PGR activity through post‐translational modifications such as phosphorylation, ubiquitination, and SUMOylation [8, 9]. These modifications can alter PGR stability, subcellular localization, and interaction with other proteins, thereby dynamically regulating PGR function.

Whilst much is known about general PGR regulation mechanisms, these cannot account for its diverse array of functions in different tissues. Colloquially known as the “pregnancy hormone,” progesterone, and its cognate receptor, PGR, are required for the differentiation and maturation of the endometrium [10], and changes in the relative expression of PGR‐A and PGR‐B isoforms are a trigger for parturition [11, 12, 13, 14]. Concurrently, PGR also mediates LH‐dependent ovulation in the ovary [15, 16, 17] and ductal elongation and side branching in mammary glands [18]. PGR also plays a role in other systemic tissues such as the central nervous system, gastrointestinal tract, and lung [19, 20]. Thus, deducing tissue‐specific regulators of PGR is vitally important for not only understanding the biology of PGR but also for the development of targeted therapies.

There have been several studies that have identified PGR interacting proteins in mammary and uterine cells [21, 22, 23, 24], however, investigations focused on PGR control of ovulation have until now relied on these mechanistic studies in unrelated tissues, and thus we have performed label‐free immunoprecipitation and mass spectrometry (IP‐MS) to identify PGR interacting proteins in the ovary utilizing the granulosa cell line KGN. Granulosa cells are the primary somatic cells in the ovarian follicle and are essential for hormone production, follicle development and oocyte quality. [25]. PGR is specifically expressed in the granulosa cells under dynamic transcriptional control by the LH surge [26]. Comparison with other PGR interactomes in other cell types [21, 22, 23, 24] demonstrates the diversity and cell specificity of PGR interacting proteins and could help uncover the mechanisms behind PGR's divergent roles.

KGN human granulosa cells were grown in Dulbecco's modified Eagle's medium/Ham's F12 nutrient mix (DMEM/F12) supplemented with 10% fetal calf serum, 0.5 M HEPES, and 100 units/mL penicillin‐streptomycin at 37°C with 10% CO_2_. Cells transduced with doxycycline‐inducible GFP‐tagged human PGR‐A (UniProt accession P06401‐2) or B (UniProt accession P06401‐1) or GFP alone as a control were selected using 1 µg/mL puromycin to enrich cells with stable expression of the transgene. PGR expression was induced by the addition of 1 µg/mL doxycycline 24 h prior to ligand treatment.

On the day of experiment, 100 nM R5020 was added to cells for 4 h. Cells were then trypsinized and 1 × 10^8^ cells were resuspended in HEPES buffered saline (40 mM HEPES pH 8.05, 150 mM NaCl) before being cross‐linked with 1 mg/mL dithiobis(succinimidyl propionate) (DSP) for 2 min with gentle rocking. 100 mM Tris‐HCl (pH 8.0) was then added to cells to quench the DSP, followed by washing in cold PBS. Cells were then lysed in mass spectrometry lysis buffer (MS buffer; 40 mM HEPES, 1% Triton X‐100, 150 mM NaCl, 2.5 mM MgCl_2_, 2 mM EDTA, 0.85% igepal/NP40, HALT protease and phosphatase inhibitors [Thermo Scientific], and 50 µM PR‐619 DUB inhibitor) at 4°C for 30 min, followed by removal of cell debris by centrifugation at 14,000 rpm for 2 min at 4°C. Protein complexes were then immunoprecipitated from the supernatants using GFP‐Trap magnetic agarose beads (ProteinTech), incubating overnight at 4°C with gentle rocking. The following day, beads were washed five times in MS lysis buffer, then once in PBS, and protein complexes eluted in 3 × 50 µL 0.1 M glycine pH 2.5, then neutralized with 30 µL of 1 M Tris‐HCl pH 9.0.

**FIGURE 1:**
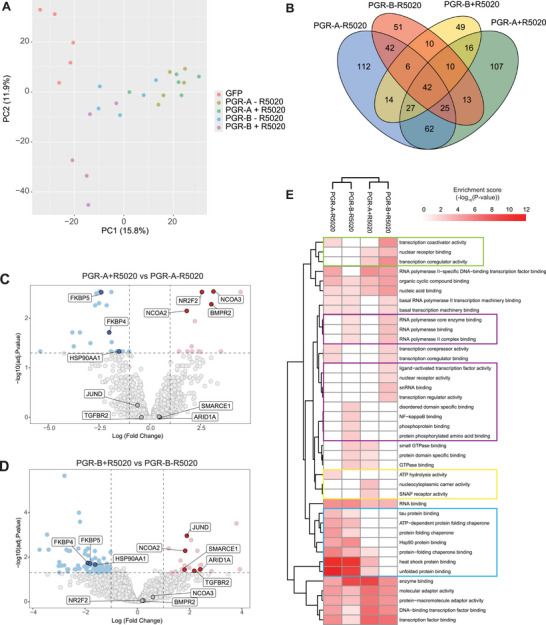
Identification of PGR‐interacting partners in the human granulosa cell line KGN. (A) Principal component analysis of replicates for each sample group. (B) UpSet plot illustrating the number of interacting proteins identified in this study and their overlap between sample groups. (C) Volcano plot showing statistical significance (‐log10 adjusted *p* value) versus magnitude of protein expression changes (log2 fold change) from a comparison of PGR‐A samples with (pink dots) and without ligand (light blue dots). (D) Volcano plot showing statistical significance (‐log10 adjusted *p* value) versus magnitude of protein expression changes (log2 fold change) from a comparison of PGR‐B samples with (pink dots) and without ligand (light blue dots). A protein was determined to be significantly differentially expressed if the log fold change (log2FC) ≥ 1 and adjusted *p* value (adj.P.val.) ≤ 0.05. Several proteins are highlighted to demonstrate interacting proteins that are common or specific to the two datasets. (E) Hierarchical clustering of gene ontology (GO) terms related to Molecular Function. The color scale represents the abundance of each GO term calculated using the *p* value ‐log transformed.

500 µg of protein complexes (*n* = 5 replicates per group: GFP alone, PGR‐A, PGR‐A+R5020, PGR‐B, and PGR‐B+R5020) were prepared for proteomic analysis using the FASP protocol previously described [27] with some minor modifications. Peptides were separated by reverse‐phase chromatography using a custom nano‐flow HPLC system (Thermo Ultimate 300 RSLC Nano‐LC, PAL systems CTC autosampler), coupled to a timsTOF Pro (Bruker) equipped with a CaptiveSpray source. The Data‐Independent Acquisition (DIA) methods were set up using the instrument firmware (timsTOF control 2.0.18.0) for data‐independent isolation of multiple precursor windows within a single TIMS scan. Data processing and analysis were performed using R (version 4.2.1). Full details on methodology can be found in Supplementary Data 1. Reproducibility of data is demonstrated by principal component analysis (Figure 1A).

We identified a total of 585 non‐redundant unique proteins that were significantly enriched in the PGR (A or B) pulldowns compared with GFP control (Figure 1B, Table S2). Initial characterization compared R5020‐dependent interactions in PGR‐A and PGR‐B pulldowns with or without R5020 treatment (Figure 1C,D). Pleasingly, as confirmation of the validity of our dataset, we identified several known PGR‐interacting proteins dependent on the treatment. For example, the chaperone proteins FKBP4, FKBP5, and HSP90 were enriched in both PGR‐A and PGR‐B pulldowns without R5020 (blue circles, Figure 1C,D), and the nuclear coactivator NCOA2 was enriched in the presence of R5020 in both PGR‐A and PGR‐B pulldowns (red circles, Figure 1C,D). We were also able to identify proteins that specifically co‐precipitated with PGR‐A or PGR‐B. For example, NCOA3, a known nuclear receptor coactivator, was specifically bound to PGR‐A in the presence of R5020, but not with PGR‐B. The orphan nuclear receptor NR2F2 was enriched with PGR‐A in the presence of R5020 (Figure 1C), whereas the AP‐1 transcription factor JUND and the SWI/SNF family members ARID1A and SMARCE1 specifically bound to PGR‐B in the presence of R5020 (Figure 1D). We also identified signaling proteins that were differentially coprecipitated dependent on the receptor isoform—for example the TGF beta receptor II (TGFBR2) preferentially coprecipitated with PGR‐B in the presence of R5020 (Figure 1D), whereas another TGF beta family member BMPR2 preferentially coprecipitated with PGR‐A in the presence of R5020 (Figure 1C).

There were many other significantly enriched proteins identified in PGR pulldowns versus GFP control as PGR interactors but were not differentially identified relative to R5020 treatment (Table S2). Examples of these include isoform‐specific interactors TRIM11, CREB1, and GRK2 (PGR‐A) and RNF40 and ADNP (PGR‐B), as well as interactors that showed no preference for specific isoforms, such as MEF2D, TOX4, and ZMIZ2. Many of these proteins have known roles in granulosa cell function or nuclear hormone receptor regulation [28, 29, 30, 31, 32, 33, 34, 35, 36], but their roles in PGR regulation are yet to be fully investigated.

Analysis of enriched GO terms with molecular function (MF) was performed on all the positively enriched proteins identified for each treatment (which includes all overlapping proteins as well as those unique to each treatment) using the functional annotation tool in The Database for Annotation, Visualization, and Integrated Discovery (DAVID) [37]. GO terms were included in the hierarchical clustering analysis when Benjamini–Hochberg FDR ≤ 0.01 (Figure 1E). Pathways related to chaperone binding and protein folding were particularly enriched in the samples without R5020 (blue box), whereas pathways relating to transcription factor activity were enriched in PGR‐A or PGR‐B expressing cells after treatment with R5020 (green box). This is consistent with the known localization of PGR proteins, which are predominantly cytoplasmic and bound to molecular chaperones in the absence of ligand but are translocated to the nucleus upon binding to ligand [2, 38]. There were only a few pathways specific to PGR‐A (gold box)—ATP hydrolysis activity was enriched in PGR‐A cells without ligand, while nucleocytoplasmic carrier activity and SNAP receptor activity were enriched in PGR‐A+R5020 treated cells. General PGR‐B specific pathways related to RNA‐polymerase binding, while the pathways specific to PGR‐B without ligand related to protein binding, including disordered domain and phosphorylated protein binding, switching to ligand activated and nuclear receptor activity after R5020 treatment (purple boxes).

**FIGURE 2:**
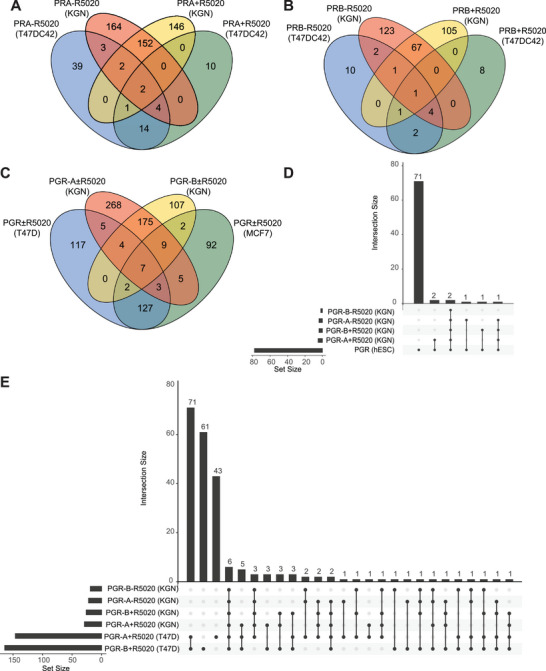
Comparative assessment of the current dataset with previously published datasets of the PGR interactome from various other cell‐types. (A and B) Venn diagrams illustrating the overlap between our dataset with that from T47DC42 cells described in Pateetin et al. [21]. (C) Venn diagram illustrating the overlap between our datasets, combining ligand treated and untreated for each isoform, and that from T47D and MCF7 cells described in Mohammed et al. [22]. (D) UpSet plot illustrating the overlap between our datasets and that from hESC cells described in Li et al. [23]. (E) UpSet plot illustrating the overlap between our dataset and that from T47D cells described in Singhal et al. [24]. In the Upset plots, only intersections containing the comparative study are illustrated.

Several previous studies have aimed to identify PGR interacting proteins [21, 22, 23, 24], however, none of these investigations use ovarian‐specific systems/models. We compared our dataset with those from other tissues to establish ovary‐specific PGR‐A and PGR‐B interactors. A list of all proteins used and their presence/absence from each dataset is listed in Table S3. Pateetin et al. expressed Tet‐inducible HA‐tagged PGR‐A or PGR‐B constructs in T47DC42 (PGR/ER negative) breast cancer cells with 10 nM R5020 treatment for 1 h [21]. Equipment used was a Q Exactive Plus mass spectrometer (Thermo Scientific) with data‐dependent acquisition (DDA) methods. Comparing the results for each isoform with our dataset demonstrated little overlap between datasets (12/537 proteins for PGR‐A, 9/324 proteins for PGR‐B; Figure 2A,B). Mohammed et al. used PR‐positive T47D and MCF7 cells with 10 nM R5020 treatment for 3 h [22]. They used a LTQ Velos‐Orbitrap MS (Thermo Scientific) coupled to an Ultimate RSLCnano‐LC system (Dionex). Specific isoform interactions were not isolated in this study, and low sample size (*n* = 2) meant no statistical significance could be calculated. Therefore, we used the full list of positive hits independent of ligand treatment and compared with our combined list of with and without ligand for each isoform (Figure 2C). Again, minimal overlap was seen between datasets (37/923 total proteins). Singhal et al. used a T47D derivative cell line with low endogenous PGR expression, reintroduced stably expressed PGR‐A or PGR‐B, and treated with 10 nM estradiol and 10 nM R5020 for 45 min [24]. An LTQ Orbitrap Velos mass spectrometer was used (Thermo Scientific) with DDA. Since they did not examine PGR interacting proteins in the absence of ligand, results were compared to all our datasets (Figure 2E). While there was a moderate degree of overlap between their treatment groups (71/218 total unique proteins), only 43/774 proteins overlapped between the two studies. Among the studies compared with our analysis, only Li et al. used primary cells, these were derived from human endometrial stroma [23]. This study treated cells with 10 nM 17β‐Estradiol and 100 µM N6,2′‐ODibutyryladenosine 3′,5′‐cyclicmonophosphate sodium salt (db‐cAMP) to induce decidualization and used 1 µM Medroxyprogesterone acetate as the PGR ligand. They used a Q Exactive Orbitrap Mass spectrometer in conjunction with a Proxeon Easy‐nLC II HPLC and nanospray source (Thermo Scientific) and also used DDA. Comparison with all our datasets revealed only 7/647 proteins in common (Figure 2D).

Despite this lack of significant overlap between studies, there were still some proteins common across multiple datasets, pointing to some generalized mechanisms of PGR control. Aside from the well characterized chaperone proteins such as HSP90 and FKBP5, the transcriptional regulators NACC1 and TMPO were both detected in 7/13 datasets, and the SH‐domain containing adaptor proteins CRKL and GRB2, as well as the deubiquitinating enzyme USP7 were detected in 6/13 datasets (Table S3). These protein‐protein interactions are largely uncharacterized and could warrant further study.

Conversely, the lack of overlap between datasets demonstrates the potential for a vast array of PGR regulatory events in different tissues. This can partially be explained by variations in methodology and experimental design, with the Mohammed et al. and Pateetin et al. studies using SILAC to identify positive hits, whereas we, Singhal et al. and Li et al. used a label free methodology. Also, our study was the one way to use data‐independent acquisition methods, where all others used data‐dependent acquisition. The use of DIA enables the detection of low‐abundance peptides that DDA might overlook, hence the overall greater number of positively identified interactors in our study. However, it would still be anticipated that there would be a greater overlap between studies with similar methodologies if those proteins were common to multiple tissues. Also, our study and the work by Pateetin et al. used inducible tagged PGR isoforms in cells lacking endogenous PGR, and Singhal et al. used PGR null cells with reintroduced stably expressed PGR‐A or PGR‐B, while the studies by Mohammed et al. and Li et al. used endogenous PGR in cells that express both isoforms. The difference in protein identification is unlikely to be attributable to endogenous versus overexpressed PGR, since the overlap between our work and the studies by Pateetin et al. or Singhal et al. was no greater than any other. Therefore, we conclude that there are a remarkable number of tissue‐specific PGR‐interacting proteins that could potentially be exploited for targeted therapeutic design.

## 2 Associated Data

The mass spectrometry proteomics data generated by the authors have been deposited in the ProteomeXchange Consortium via the PRIDE partner repository [39] with the dataset identifier PXD056566. Proteomics datasets from published studies that were used in comparisons are deposited in the PRIDE repository (PXD02392036 [21], PXD002104 [22]) or in the MassIVE repository (MSV000092346 [23]). Singhal et al. did not describe any deposit of their raw data, but summary files were found in the Supplementary Materials of their paper [24].

## Conflicts of Interest

The authors have declared no conflict of interest.

## Supporting information



Supporting Information

Supporting Information

Supporting Information

Supporting Information

## Data Availability

The authors have nothing to report.
