# Supplementary material for: Comparative Analysis of the Progesterone Receptor Interactome in the Human Ovarian Granulosa Cell Line KGN and Other Female Reproductive Cells
Source: Proteomics. 2025 May 13;25(13):e202400374. doi: 10.1002/pmic.202400374 (PMC12246768; doi:10.1002/pmic.202400374)
Supplement: Supplementary file 1 — Supporting Information [file PMIC-25-e202400374-s001.docx]

**Supplementary Data 1**

**Supplementary methods**

**Generation of PGR constructs**

Doxycycline-inducible N-terminal GFP-tagged full length PGR-A or B (or GFP alone as control) was cloned into the lentiviral vector pLV-TRE3G-GtwyA-EF-Tet-On3G-P2A-puro (gifted from David Bersten, University of Adelaide, Adelaide, Australia) using the Gibson assembly method. [1]

**Sample preparation for mass spectrometry**

Eluates from immunoprecipitations were prepared for LC-MS/MS analysis using the Filter Assisted Sample Preparation (FASP) method [2, 3]with the following modifications. Briefly, eluates were added to a Vivacon® 30 kDa MWCO (Sartorius, VN01H22), and proteins were solubilised and reduced in 8M Urea in 100 mM Tris-HCl (pH 7.5) containing 10 mM Tris-(2-carboxyethyl)phosphine (TCEP, Sigma) for 30 min, alkylated with 50 mM iodoacetamide (Sigma), then digested with 1 μg Trypsin (SoLuTrypsin, Sigma) after buffer exchange to 50 mM ammonium bicarbonate (AmBic) and incubated overnight at 37 °C. Peptides were eluted with 50 mM AmBic in two 75 μl sequential washes and acidified in 1% formic acid (FA, final concentration). Peptides were lyophilized to dryness using a CentriVap (Labconco) before being reconstituted in 0.1% FA/2% ACN ready for mass spectrometry analysis.

**LC MS/MS analysis and data analysis** Peptides (1 μL) were separated by reverse-phase chromatography on a C18 fused silica column (inner diameter 75 μm, OD 360 μm × 15 cm length, 1.6 μm C18 beads) packed into an emitter tip (IonOpticks) using a custom nano-flow HPLC system (Thermo Ultimate 300 RSLC Nano-LC, PAL systems CTC autosampler). The HPLC was coupled to a timsTOF Pro (Bruker) equipped with a CaptiveSpray source. Peptides were loaded directly onto the column at a constant flow rate of 400 nL/min with buffer A (99.9% Milli-Q water, 0.1% FA) and eluted with a 30-min linear gradient from 2 to 34% buffer B (90% ACN, 0.1% FA). The timsTOF Pro (Bruker) was operated in diaPASEF mode using Compass Hystar 5.1. The settings on the TIMS analyzer were as follows: Lock Duty Cycle to 100% with equal accumulation and ramp times of 100 ms, and 1/K0 Start 0.6 V.·/cm2 End 1.6 V·s/cm2, Capillary Voltage 1400V, Dry Gas 3 l/min, Dry Temp 180°C. The Data-Independent Acquisition (DIA) methods were set up using the instrument firmware (timsTOF control 2.0.18.0) for data-independent isolation of multiple precursor windows within a single TIMS scan. The method included two windows in each diaPASEF scan, with window placement overlapping the diagonal scan line for doubly and triply charged peptides in the m/z – ion mobility plane across 16 × 25 m/z precursor isolation windows (resulting in 32 windows) defined from m/z 400 to 1,200, with 1 Da overlap, and CID collision energy ramped stepwise from 20 eV at 0.8 V·s/cm 2 to 59eV at 1.3 V·s/cm2. DIA data were analysed using DIA-NN 1.8 in library-free mode [4]. Bruker diaPASEF d. files were searched against reviewed sequences from Human Uniprot Reference Proteome (downloaded July 2022) with the following settings: trypsin specificity, peptide length of 7-30 residues, cysteine carbidomethylation as a fixed modification, variable modifications set to n-terminal protein acetylation and oxidation of methionine, the maximum number of missed cleavages at 2. Mass accuracy was set to 10 ppm for both MS1 and MS2 spectra and match between runs (MBR) enabled, and filtering outputs set at a precursor q-value < 1%.

**Data processing**

Proteins without any proteotypic precursors or with q-value greater than 0.01 or identified by a single peptide were removed. A further filtration step was done, where proteins identified in 60% or more of samples in one group were kept. A total of 5,374 proteins were included in the analysis. Protein intensities were log2-transformed and normalised using RUVIIIC (v. 1.0.19). Invariant proteins in all conditions (P-value > 0.5), with coefficient of variation (CV%) < 2%, were chosen as negative controls for RUVIIIC normalisation. Missing values were imputed by applying Barycenter approach for Missing Not At Random (v2-MNAR) method implemented in msImpute package (v. 1.7.0). Differential analysis was performed using limma (v. 3.52.4). A protein was determined to be significantly differentially expressed if the false discovery rate (FDR) was ≤ 0.05 after Benjamini–Hochberg correction.

Analysis of enriched Gene Ontology (GO) terms associated with Molecular Function (MF) was performed on all the positively enriched protein identified for each treatment (which includes all overlapping proteins as well as those unique to each treatment) using the functional annotation tool in The Database for Annotation, Visualization and Integrated Discovery (DAVID). [5] GO terms were included when Benjamini–Hochberg FDR ≤ 0.01, which were -log10 transformed for heatmap generation using pheatmap package in R software (Table S1, Figure 1D).

[1] Gibson, D. G., Young, L., Chuang, R. Y., Venter, J. C.*, et al.*, Enzymatic assembly of DNA molecules up to several hundred kilobases. *Nat Methods* 2009, *6*, 343-345.

[2] Sharma, R., Dill, B. D., Chourey, K., Shah, M.*, et al.*, Coupling a detergent lysis/cleanup methodology with intact protein fractionation for enhanced proteome characterization. *J Proteome Res* 2012, *11*, 6008-6018.

[3] Wisniewski, J. R., Zougman, A., Nagaraj, N., Mann, M., Universal sample preparation method for proteome analysis. *Nat Methods* 2009, *6*, 359-362.

[4] Demichev, V., Szyrwiel, L., Yu, F., Teo, G. C.*, et al.*, dia-PASEF data analysis using FragPipe and DIA-NN for deep proteomics of low sample amounts. *Nat Commun* 2022, *13*, 3944.

[5] Sherman, B. T., Hao, M., Qiu, J., Jiao, X.*, et al.*, DAVID: a web server for functional enrichment analysis and functional annotation of gene lists (2021 update). *Nucleic Acids Res* 2022, *50*, W216-W221.
